# Functionalized MoS_2_-nanoparticles for transdermal drug delivery of atenolol

**DOI:** 10.1080/10717544.2020.1778815

**Published:** 2020-06-29

**Authors:** Kai Zhang, Yanling Zhuang, Weidan Zhang, Yali Guo, Xiaochang Liu

**Affiliations:** a College of Science and Technology, Hebei Agricultural University, Cangzhou, China; b College of Humanities and Management, Hebei Agricultural University, Cangzhou, China; c Department of Cardiology and Nephrology, Rongcheng County People’s Hospital, Xiongan, China; d School of Pharmacy, Shenyang Medical College, Shenyang, China; e Translational Medicine Research Centre, Shenyang Medical College, Shenyang, China

**Keywords:** Molybdenum disulfide, transition-metal dichalcogenides, cationic hydroxyethyl cellulose, transdermal drugs, drug release, biomedicine

## Abstract

Molybdenum disulfide (MoS_2_) has excellent photothermal conversion abilities, an ultra-high specific surface area, and has been extensively explored for use in biomedicine. However, the high toxicity associated with MoS_2_ has limited its biological applications for *in vivo* photothermal therapy and drug delivery systems. Herein, we have developed cationic hydroxyethyl cellulose (JR400) surface-modified MoS_2_ nanoparticles (NPs) that are responsive to near-infrared (NIR) laser irradiation as a transdermal drug delivery system (TDDS). Herein, we confirmed the preparation of hexagonal phase MoS_2_ with robust surface modification with JR400. The flower-like morphology of the NPs had an average diameter of 355 ± 69.3 nm limiting the absorption of the NPs through the stratum corneum. With the ability to efficiently load 90.4 ± 0.3% of the model drug atenolol (ATE), where 1 g of JR400-MoS_2_ NPs was able to load 3.6 g ATE, we assayed the controlled release capacity *in vitro* skin penetration studies. These JR400-MoS_2_ NPs showed further enhancement under NIR stimulation, with a 2.3-fold increase in ATE skin penetration. Furthermore, we verified *in vivo* that these JR400-MoS_2_ NPs do not cause skin irritation suggesting that they are promising new TDDS candidates for small molecule drugs.

## 1. Introduction

Molybdenum disulfide (MoS_2_) is a typical transition-metal dichalcogenide with a layered structure consisting of individual S-Mo-S layers weakly bound by Van der Waals forces (Chen et al., 2015; Dhall et al., 2015; Wang et al., 2019). The remarkable physicochemical properties of MoS_2_ has led to extensive research and applications in a wide variety of fields, including photochemistry, optoelectronics, catalysis, hydrogen storage, and biomedicine (Zhu et al., 2013; Qiao et al., 2016; Zhang et al., 2016). MoS_2_ has been widely investigated for applications in drug delivery systems because of its ultra-high specific surface area (Liu et al., 2014; Yin et al., 2014; Wang et al., 2015; Wu et al., 2018; Zhang et al., 2018). Liu et al. used MoS_2_ nanosheets paired with a PEG carrier to load a variety of therapeutic molecules, where the drug loading ratio of the MoS_2_ nanosheets was superior to graphene oxide (Liu et al., 2014). Additionally, the release of the loaded drug molecules from the MoS_2_ is inducible using 808-nm near-infrared (NIR) laser irradiation (Yin et al., 2014). The excellent photothermal performance of MoS_2_ has inspired further use in photothermal therapies (Wang et al., 2015; Liu et al., 2016; Huang et al., 2017; Zhang et al., 2017; Fu et al., 2018). Wang et al. evaluated a MoS_2_/Bi_2_S_3_ composite as a photothermal therapy agent (Wang et al., 2015). Chou et al. developed MoS_2_-based NIR photothermal agents comprising Ce-MoS_2_ nanosheets that offered higher photothermal performance than graphene and gold nanorods (GNRs) (Chou et al., 2013). However, the toxicity of MoS_2_ has presented challenges for its use in photothermal therapy and drug delivery *in vivo*. Yu et al. found that 20 mg/L chitosan-functionalized MoS_2_ micro-sheets exhibited cytotoxicity in the gills and liver of adult zebrafish (Yu et al., 2018). Furthermore, nano-MoS_2_ and MoS_2_ nanosheets exfoliated with t-Bu-Li and n-Bu-Li have been found to be cytotoxic (Chng et al., 2014; Wu et al., 2019). To date, the *in vivo* toxicity of MoS_2_ remains a critical barrier for its use in biomedical applications.

Toxicity of MoS_2_ can be mitigated by developing biomedical applications that can be used *ex vivo*. Transdermal drug delivery systems (TDDSs) are an innovative approach to delivering drugs into the blood at a controlled rate via the skin. TDDSs avoid the first-pass effect and improve patient compliance because they are applied topically (Labouta et al., 2011; Anselmo & Mitragotri, 2014; Pastore et al., 2015; Liu et al., 2017a, 2017b; Charoensumran & Ajiro, 2020). Materials larger than 45 nm are stopped at the stratum corneum (SC) of untreated intact human skin, allowing for sustained and controlled drug release (Labouta et al., 2011). MoS_2_ is expected to be an outstanding TDDS material, but, to the best of our knowledge, has yet to be reported in the literature.

Herein, we aimed to overcome the toxicity of MoS_2_ in biomedical applications by developing an innovative TDDS using three-dimensional (3D) flower-like MoS_2_ nanoparticles (NPs) produced via a simple hydrothermal approach (Zhang et al., 2015). Cationic hydroxyethyl cellulose (JR400) was electrostatically bound to the negatively charged surface of the MoS_2_ NPs, increasing its colloidal stability and biocompatibility (Liu et al., 2006; Ran et al., 2019). The MoS_2_ NPs coated with JR400 (JR400-MoS_2_ NPs) were further characterized using attenuated total reflection Fourier transform infrared (ATR-FTIR) spectroscopy, X-ray diffraction (XRD), and transmission electron microscopy (TEM). We chose to load the J400-MoS_2_ NPs with the model drug atenolol (ATE), a β_1_-adrenergic receptor blocking agent prescribed for hypertension. The drug load efficacy and photothermal conversion effect were evaluated, and the controlled release capacity of JR400-MoS_2_ NPs was demonstrated in *in vitro* drug release and skin penetration assays.

## 2. Materials and methods

### 2.1. Materials

Ammonium molybdate tetrahydrate and thiourea were obtained from Shanghai Macklin Biochemical Co. Ltd. (Shanghai, PR China). Cationic hydroxyethyl cellulose (JR400, Mw = 8,000,000 g/mol) was purchased from Shandong Usolf Chemical Technology Co. Ltd. (Linyi, PR China). Atenolol (HPLC grade, 98%) was purchased from Rhawn Chemical Technology Co. Ltd. (Shanghai, PR China). HPLC grade methanol and phosphoric acid were used throughout. All other chemicals were reagent grade and obtained commercially. All chemicals were used as received without any further purification. Ultrapure water was purified by a Milli-Q system (18.2 MΩ).

### 2.2. Synthesis of JR400-MoS_2_ NPs

A facile and straightforward hydrothermal method was used to synthesize flower-like MoS_2_ NPs (Zhang et al., 2015). In detail, 1.24 g of ammonium molybdate tetrahydrate and 2.28 g of thiourea were dissolved in 36 mL of ultrapure water under vigorous stirring for 30 min to form a homogeneous solution. The mixture was transferred into a 50-mL Teflon-lined stainless-steel autoclave and heated at 220 °C for 6 h. After unassisted cooling to room temperature, the products were collected by centrifugation and washed with ultrapure water and absolute ethanol several times, then dried in a vacuum at 60 °C.

Afterward, 1.0 g of the obtained flower-like MoS_2_ NPs was stirred into 200 mL of aqueous containing 0.2 g of JR400. The suspension was ultrasonicated for 1 h, then heated at 80 °C for 4 h. The product was separated by centrifugation and then washed with deionized water and absolute ethanol several times. After vacuum drying at 60 °C for 24 h, we obtained the JR400-MoS_2_ NPs (black powder).

### 2.3. Characterization of JR400-MoS_2_ NPs

Spectrum of JR400-MoS_2_ NPs was collected using the KBr pellets method with ATR-FTIR spectroscopy (Perkin Elmer 2000, ‎Waltham, MA). The crystalline structure of JR400-MoS_2_ NPs was studied using Rigaku SmartLab X-Ray Diffraction with Cu Kα radiation. The microstructure of the samples was investigated using TEM (JEOL-2100F, Akishima, Japan), which was operated at 200 kV. The particle size and zeta potential were measured using dynamic light scattering (DLS, Melvin 2000). The colloidal stability was analyzed by zeta potential and sedimentation volume ratio. The sedimentation volume ratio was determined by the ratio of the height after (*H*_u_) and before (*H*_0_) the sedimentation.

### 2.4. Photothermal conversion performance

JR400-MoS_2_ NPs were suspended in water at varying concentrations from 0.1 to 1.0 mg/mL for irradiation with an 808-nm laser (0.5 W/cm^2^) to measure the photothermal effects. The laser power density was also varied between 0.2 and 1.0 W/cm^2^. The thermal stability of the JR400-MoS_2_ NPs at 0.5 mg/mL was determined by NIR-stimulation (0.5 W/cm^2^) for 5 min over three on-off cycles. The temperature of the solution was measured by a magnetic stirrer equipped with a temperature probe.

### 2.5. Drug loading

ATE was loaded onto the surface of JR400-MoS_2_ NPs by mixing different concentrations of the drug with 0.5 mg/mL JR400-MoS_2_ NPs in 5 mL of phosphate-buffered saline (PBS) (pH = 7.0). The mixture was stirred for 2 h and incubated at 32 °C for 24 h. The free drug was removed by centrifugation at 5000 r/min for 10 min, and the JR400-MoS_2_-ATE NPs were washed with ultrapure water three times. The JR400-MoS_2_-ATE NPs were dried at 60 °C for 24 h under vacuum and stored at room temperature. The drug loading was calculated by the concentration of free drug in the supernatant, as analyzed by HPLC. Drug loading efficiency and drug loading percent were calculated using the following equations:

$$\text{Drug loading efficiency}=\frac{\text{the amount of loading drug}}{\text{the amount of NPs }}\times 100\%$$

$$\text{Drug loading percent}=\frac{\text{the amount of loading drug}}{\text{the amount of total drug}}\times 100\%$$

### 2.6. *In vitro* drug release and skin permeation experiments

#### 2.6.1. Preparation of the donor solution

A donor solution was prepared by adding 25 mg JR400-MoS_2_-ATE into 50 mL PBS. In order to form a uniform suspension, the mixture was stirred for 1 h, followed by sonication for 15 min. The suspension was added into the donor chamber of a two-chamber diffusion cell immediately after preparation.

#### 2.6.2. *In vitro* drug release experiments

Drug release was assayed using a two-chamber diffusion cell separated by a 0.22-μm cellulose microporous membrane. 4.0 mL of JR400-MoS_2_-ATE solution was added into the donor chamber, and an equal volume of PBS (pH 7.4) containing 15% PEG400 (PBS-PEG400) was added into the receptor chamber. Both chambers were continuously stirred with a magnetic stirrer at 600 rpm and kept at 32 °C. 2.0 mL from receptor chamber was collected at 1, 2, 3, 4, 5, 6, 7, 8, 12, 24, 28, 32, 36, and 48 h, and then replaced with the same volume of fresh PBS-PEG400. The donor solution was irradiated with laser (0.5 W/cm^2^) for 5 min after sampling.

#### 2.6.3. *In vitro* skin permeation experiments

Male Wistar rats (180–220 g, 6–8 weeks old) were supplied by Liaoning Changsheng (Liaoning, China). Full-thickness skin was prepared as follows: (1) the rat was anesthetized with urethane (20% w/v, 6 mL/kg, i.p.), and the abdominal hair was shaved off. (2) Abdominal skin was excised after the rat was sacrificed. (3) The adhering subcutaneous tissues were carefully removed. All the procedures were performed in accordance with the NIH Guidelines for the Care and Use of Laboratory Animals and were approved by the Animal Ethics Committee of Shenyang Medical College.

The two-chamber diffusion cell was used for the permeation experiments. The skin was mounted between the two chambers with SC facing the donor solution and epidermis facing the receptor solution. Sample collection and drug concentration measurements were performed in the same manner as drug release study, except that sample collection was conducted at 2, 4, 6, 8, 12, 24, 28, 32, 36, and 48 h with NIR-stimulation after every sampling. The cumulative drug skin penetration amount was calculated using the drug concentration in the receptor solution, taking the effect of sampling into account.

#### 2.6.4. HPLC analysis of the drug

The drug concentration was determined by Hitachi HPLC (Tokyo, Japan), which consisted of a Pump L-2130, AutoSampler L-2200, UV-detector L-2420, and C18 reversed-phase column (200 × 4.6 mm, 5 μm, ODS-2 Waters, Milford, MA). The mobile phase for the ATE was a mixture of methanol, water, and phosphoric acid solution (70:30:0.1, v/v). The column temperature was 25 °C, the flow rate was set at 0.7 mL/min, and the free drug was detected at a wavelength of 275 nm.

### 2.7. *In vivo* skin erythema study

A Mexameter^®^ (MX 16, Courage & Khazaka Co., Cologne, Germany) was used to measure the biocompatibility of JR400-MoS_2_ NPs by measuring the erythema index (EI) of the skin. Rabbits were used to assess potential skin irritation caused by JR400-MoS_2_ NPs compared with a 10% (w/v) aqueous solution of sodium dodecyl sulfate (SDS) as the positive control. The dorsal skin of the rabbits was shaved and divided into three separate sections, each with an area of 2.5 cm × 2.5 cm. The initial EI values of the chosen sections were measured as a baseline value (EI_0_) before topical application of 500 μL of PBS, JR400-MoS_2_ NPs, or 10% SDS in their respective sections. The application sites were covered with double-layer gauze in order to prevent perturbation. After 8 h, the excess solutions were removed, and the predefined sections were gently cleaned with cotton wool swabs. The EI_t_ was measured at set intervals and ΔEI was calculated by subtracting EI_0_ from EI_t_. This experiment was performed in quadruplicate.

### 2.8. Statistical analysis

Results are reported as the mean ± SD. The data were subjected to analysis of variance (ANOVA) using the SPSS 16.0 software (SPSS Inc., Chicago, IL). Significance levels are reported for *p*< .05.

## 3. Results and discussion

### 3.1. Synthesis and characterization of JR400-MoS_2_ NPs

**Figure 1.**
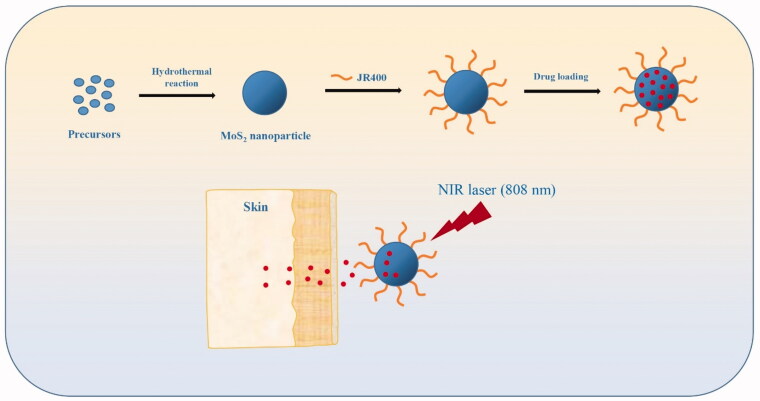
Schematic showing the assembly of JR400-MoS_2_ NPs and their application for the controlled release of ATE via transdermal administration.

Flower-like MoS_2_ NPs were synthesized using a previously reported hydrothermal process using ammonium molybdate tetrahydrate and thiourea precursors to produce polyacrylamide (PAM) modified MoS_2_ NPs (Wang et al., 2019). NPs were subsequently surface modified with JR400 (Figure 1), a water-soluble cellulose derivative that is currently used in skincare products (Li et al., 2012).

**Figure 2.**
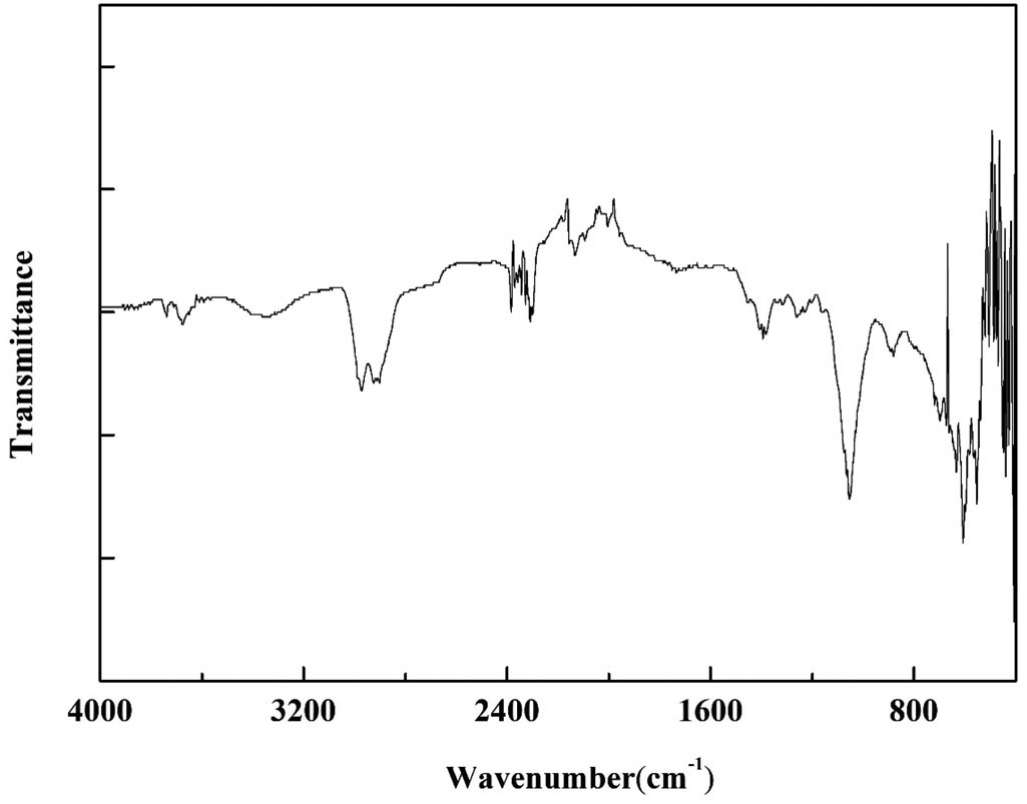
ATR-FTIR spectra of JR400-MoS_2_ NPs.

The JR400 surface modification was evaluated using ATR-FTIR spectroscopic analysis (Figure 2). The peaks present at 2875 cm^–1^, 2974 cm^–1^, and 3409 cm^–1^ were attributed to the symmetrical stretching vibrations of the C–H bond, asymmetrical stretching vibrations of the C–H bond, and stretching vibration of the O–H band from the JR400 molecule, respectively, indicating that the MoS_2_ NPs were readily surface-modified with JR400.

**Figure 3.**
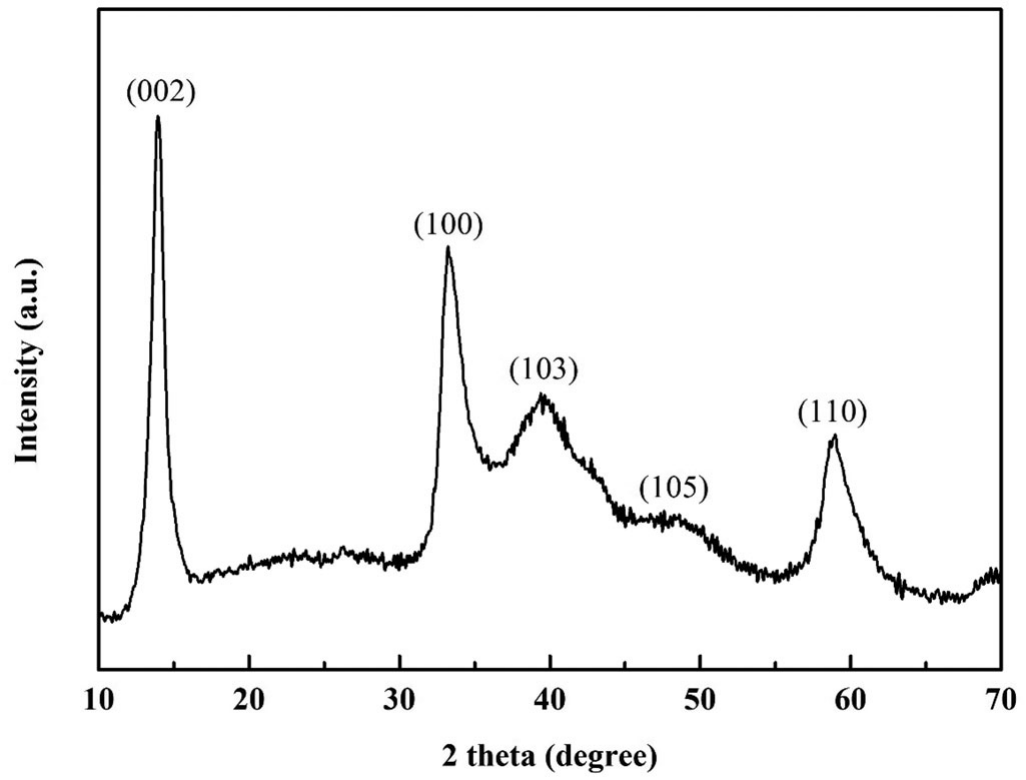
The XRD pattern of the JR400-MoS_2_ NPs corresponding with the hexagonal phase of MoS_2_.

The XRD patterns exhibited five well-resolved diffraction peaks at 2*θ* = 14.4°, 32.7°, 39.5°, 49.8°, and 58.3°, which can be assigned to the (002), (100), (103), (105), and (110) diffraction planes, respectively (Figure 3). These planes correspond to the hexagonal phase of MoS_2_ (JCPDS no. 37-1492) (Tang et al., 2013). No other diffraction peaks were observed; thus, a hexagonal phase of MoS_2_ was prepared.

**Figure 4.**
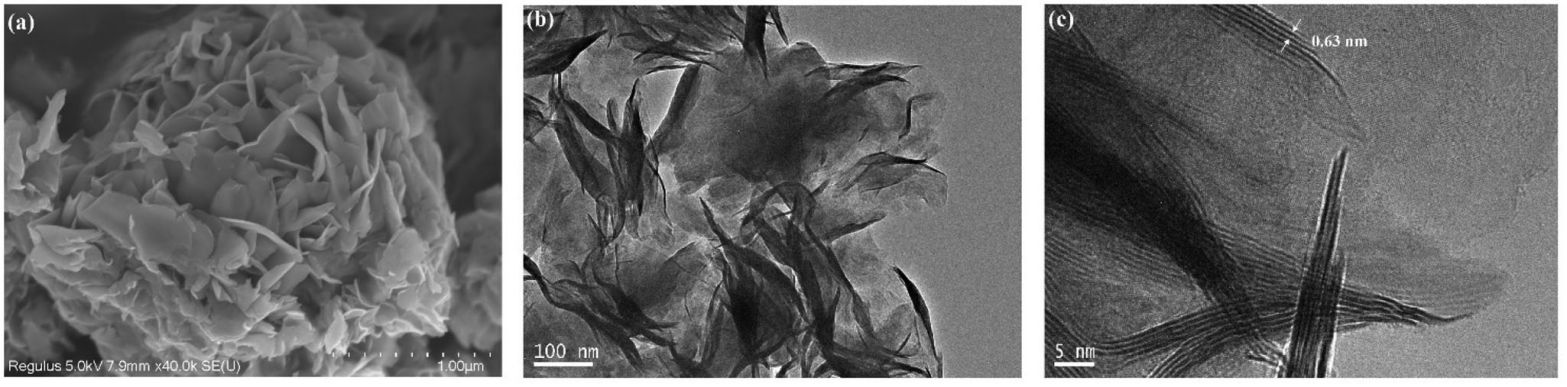
(a) SEM, (b) TEM, and (c) HRTEM images were used to visualize the grain size, morphology, and structure of the JR400-MoS_2_ NPs.

The grain size, morphology, and structure of the JR400-MoS_2_ NPs were investigated using scanning electron microscopy (SEM), TEM, and high-resolution TEM (HRTEM) (Figure 4). The SEM images indicated that the JR400-MoS_2_ NPs formed via irregular curling and winding of the thin MoS_2_ nanosheets (Figure 4(a)). The structure was studied in more detail using TEM and HRTEM. TEM revealed the NPs had a flower-like morphology (Figure 4(b)), consistent with the SEM images. HRTEM showed that the NPs were made from overlapping MoS_2_ nanosheets, matching previous reports in the literature (Tang et al., 2012). The lattice spacing of the MoS_2_ (002) plane was observed in the enlarged HRTEM image (Figure 4(c)), where the 0.63-nm lattice spacing was consistent with the XRD results.

The JR400-MoS_2_ NPs had an average diameter of 355 ± 69.3 nm (PDI = 0.259), as obtained by DLS, similar to the size obtained by HRTEM image (Figure 4(c)). Generally, particles larger than 45 nm are stopped by the SC of skin (Labouta et al., 2011). Thus, the potential *in vivo* toxicity of JR400-MoS_2_ NPs is significantly reduced as it cannot translocate into the bloodstream. Furthermore, the JR400-MoS_2_ NPs could be used in TDDS for the controlled release of the drug across the SC.

**Figure 5.**
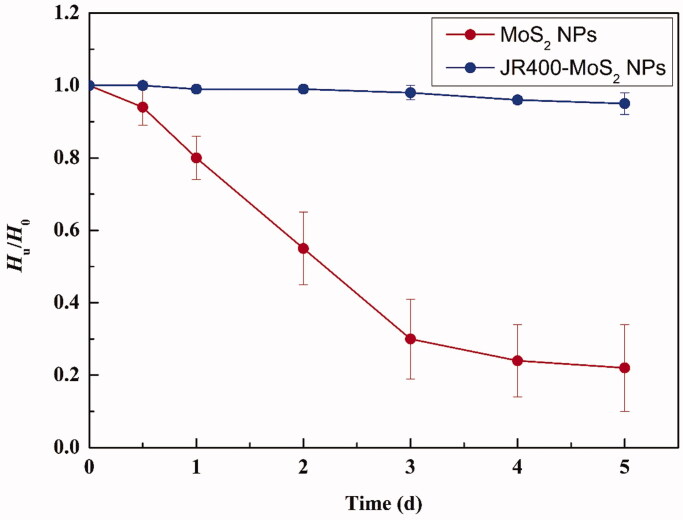
The colloidal stability of JR400-MoS_2_ NPs: sedimentation volume ratio (*H*_u_/*H*_0_) versus time.

The colloidal stability of JR400-MoS_2_ NPs and MoS_2_ NPs was explored using zeta potential, sedimentation volume ratio, and dispersion times. The zeta potential of the MoS_2_ NPs was measured to be –14.64 ± 1.72 mV, while the zeta potential of JR400 MoS_2_ NPs was found to be significantly more negative at –25.52 ± 2.15 mV, indicating that the JR400 functionalization increases the stability of the particle (Khafaji et al., 2019). After five days of sedimentation, the *H*_u_/*H*_0_ of JR400-MoS_2_ NPs and MoS_2_ NPs were 0.95 ± 0.03 and 0.22 ± 0.12, respectively (Figure 5). The results were probably related to hydroxyl groups in JR400, which could form hydrogen bond with water. Overall, modifying the surface of the MoS_2_ NPs with JR400 made them more stable in water.

### 3.2. Photothermal conversion performance study

**Figure 6.**
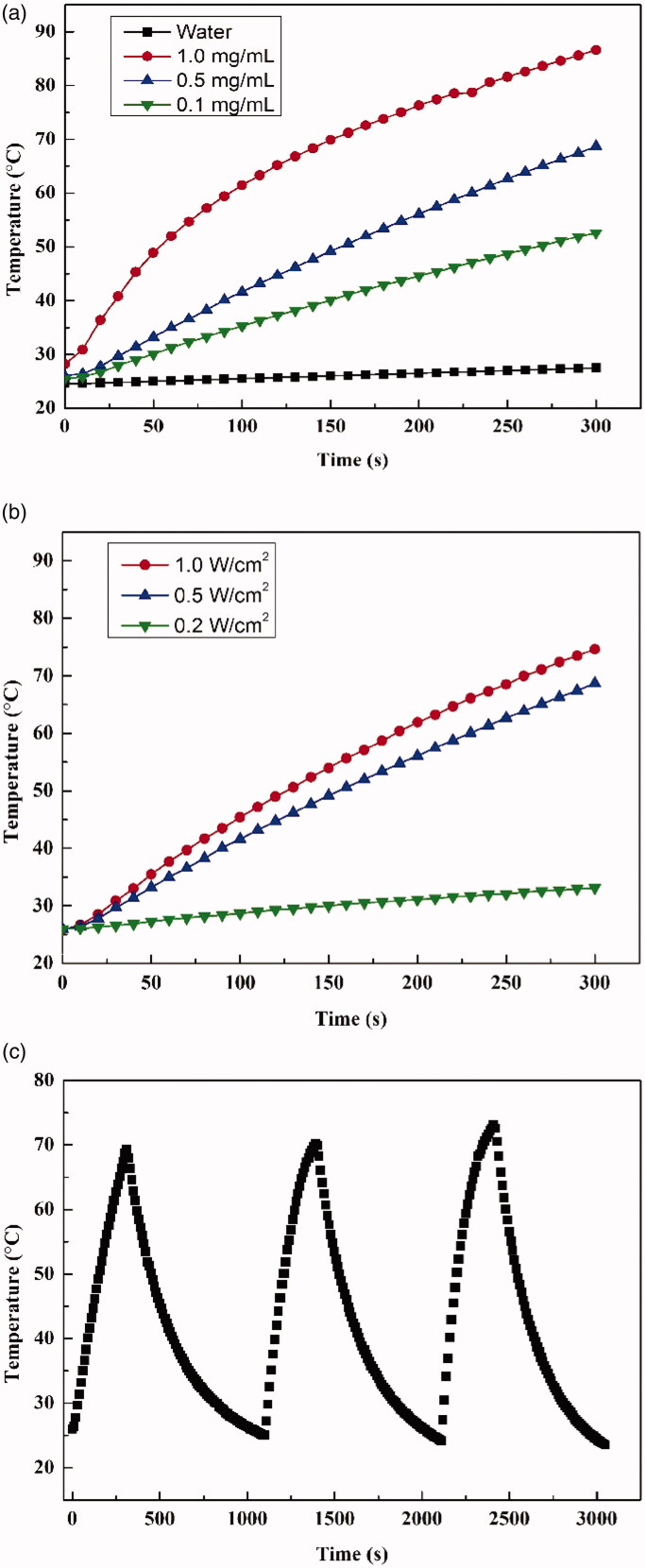
The heating curves of (a) JR400-MoS_2_ NPs of various concentrations under laser power of 0.5 W/cm^2^, (b) 0.5 mg/mL JR400-MoS_2_ NPs treated with varying laser power, and (c) temperature of the JR400-MoS_2_ NP solution (0.5 W/cm^2^, 0.5 mg/mL) over three laser cycles.

The photothermal properties of NP drug carriers have been reported to have a substantial effect on drug release (Yin et al., 2014). Previous studies have shown MoS_2_ exhibits absorption at 808 nm (Yang et al., 2018). The conversion efficiency of the JR400-MoS_2_ NPs was studied by changing the power density of the laser and concentration of the MoS_2_ NPs. The temperature of the 1.0 mg/mL JR400-MoS_2_ NP solutions increased rapidly to 86.6 °C during the 5 min of irradiation, while the control samples exhibited minimal changes under the same conditions (Figure 6(a)). The temperature increase was dependent on the concentration of NPs, as well as the power density of the laser (Figure 6(a,b)). Considering the enhanced effect on drug release and overall skin irritation, an NP concentration of 0.5 mg/mL and a power density of 0.5 W/cm^2^ were used for the *in vitro* experiments. The conversion stability was confirmed by conducting three on–off cycles (Figure 6(c)). Overall, the JR400-MoS_2_ NPs exhibited outstanding photothermal conversion ability.

### 3.3. Drug loading

**Figure 7.**
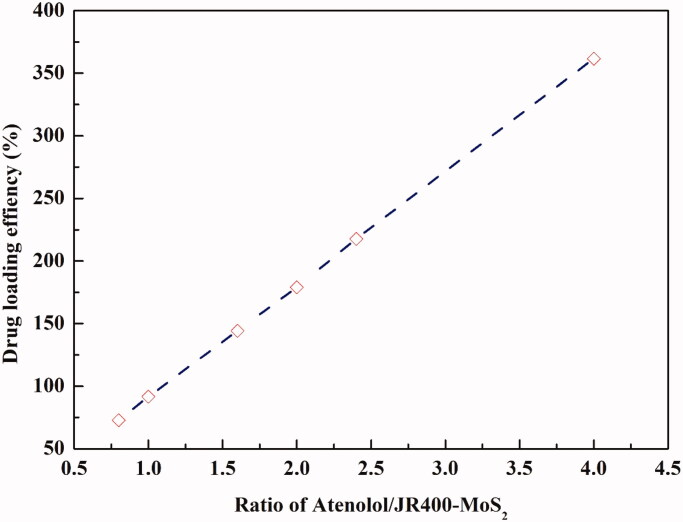
Drug loading efficiency achieved as a function of the mass ratio of ATE to JR400-MoS_2_ NPs.

MoS_2_ nanosheets have been used as drug delivery systems, but the multilayer structure has led to compromised drug loading efficiency. The porous structure of the 3D flower-like MoS_2_ increases the number of drug loading sites (Yang et al., 2018). Using ATE as a model drug, higher drug concentrations yielded greater loading, with a 4:1 mass ratio of ATE to JR400-MoS_2_ NPs led to optimal drug loading efficiency (Figure 7). At this ratio, 1.0 g of JR400-MoS_2_ NPs was able to load 3.6 g of ATE, resulting in a drug loading efficiency of 361.6 ± 1.0% with 90.4 ± 0.3% of the initial drug loaded onto the NPs. An optimized concentration of 0.5 mg/mL JR400-MoS_2_ NPs with 2.0 mg/mL ATE was selected.

### 3.4. Drug release experiments

**Figure 8.**
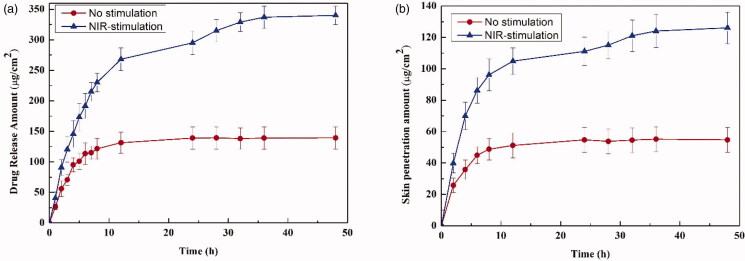
(a) *In vitro* drug release of ATE from JR400-MoS_2_ NPs with (blue) and without (red) NIR stimulation. The concentration of ATE was determined via diffusion through a 0.22 μm cellulose microporous membrane. (b) *In vitro* skin penetration of ATE released from the JR400-MoS_2_ NPs with (blue) and without (red) NIR stimulation.

The release of ATE from the JR400-MoS_2_ NPs was investigated using a classic transdermal drug release study with a two-chamber diffusion cell (Sun et al., 2012). Without NIR irradiation of the JR400-MoS_2_ NPs, only 139.4 ± 18.17 μg/cm^2^ ATE was released within 48 h (Figure 8(a)). However, NIR-stimulation at 0.5 W/cm^2^ for 5 min caused a significant increase releasing 340.12 ± 17.84 μg/cm^2^ of the drug, indicating that NIR stimulation was beneficial for the release of ATE. Moreover, the use of NIR-stimulation significantly increased the release of ATE in 36 h. These results demonstrate the potential for a controllable JR400-MoS_2_ NP drug delivery system.

Nanoparticle drug delivery systems commonly use NIR stimulation to facilitate drug release. Shao et al. presented a polymeric microcapsule drug delivery system involving water-soluble antitumor drug encapsulation and GNR functionalization, where low power NIR radiation was applied to trigger the release of the drug (Shao et al., 2015). Similarly, Koning et al. developed a thermosensitive liposome drug delivery system that released the drug upon hyperthermia (Koning et al., 2010). The drug loading capacity of the JR400-MoS_2_ NPs was high in comparison with these other reported NPs and is expected to allow for a more sustained release of drugs.

### 3.5. Skin penetration study

An *in vitro* skin penetration assay is one way to predict drug penetration *in vivo* and has been validated by *in vitro*–*in vivo* correlation studies (Elmowafy et al., 2019). The amount of skin penetration in the NIR-stimulated group was 125.11 ± 8.58 μg/cm^2^, which was 2.3 times higher than the control, indicating that JR400-MoS_2_ NPs are suitable for TDDS applications (Figure 8(b)).

Transdermal penetration of small molecule drugs involves two critical steps: (1) drug release from the matrix and (2) percutaneous absorption. JR400-MoS_2_ NPs were not able to pass through the intact skin because of their relatively large particle, but they can easily control drug released into the matrix. Intercellular lipids of the SC are the main barrier to the penetration of drugs across the skin. The diffusion coefficient of intercellular lipids of SC could be increased by rising temperature of the skin, which could increase the mobility of the intercellular lipid (Liu et al., 2017). JR400-MoS_2_ NPs showed excellent photothermal conversion ability and an increased diffusion coefficient for both the drug and the intercellular lipids, resulting in increased percutaneous drug absorption (Haine et al., 2017; Teodorescu et al., 2017).

### 3.6. *In vivo* skin erythema study

**Figure 9.**
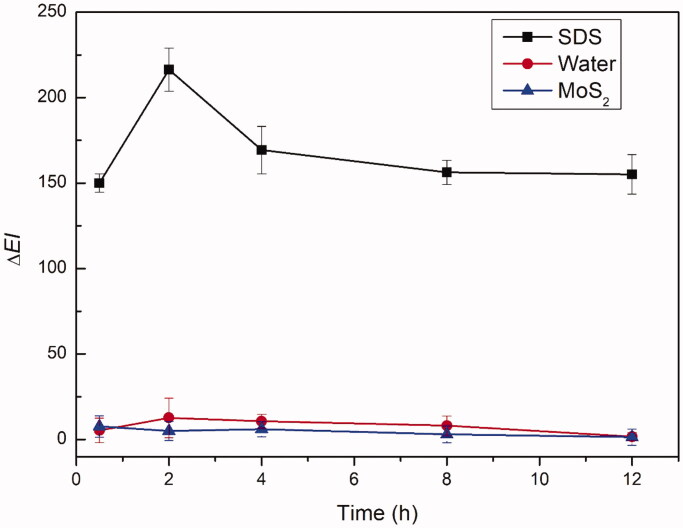
Biocompatibility of JR400-MoS_2_ NPs: *in vivo* skin erythema study of 10% SDS, water, and JR400-MoS_2_ NPs.

Visual observation of erythema is widely used to evaluate the skin irritant potential of substances. However, this method has been criticized as imprecise and subjective. A noninvasive *in vivo* skin erythema measurement was used to monitor irritation caused by the JR400-MoS_2_ NPs. As shown in Figure 9, the ΔEI increased significantly after the topical application of 10% SDS, serving as an established positive control. Application of the JR400-MoS_2_ NPs suspended in water did not increase Δ*EI*, indicating high biocompatibility.

## 4. Conclusions

JR400-functionalized MoS_2_ NPs were synthesized as a TDDS for ATE, a β_1_-adrenergic receptor blocking agent prescribed for hypertension. The ultra-high specific surface area of the JR400-functionalized MoS_2_ NPs allowed for efficient binding of ATE with 90% loading efficiency. NIR stimulation enhanced drug release and facilitated drug skin penetration. The application of JR400-MoS_2_ NPs as a TDDS shows the potential to mitigate issues associated with the toxicity of MoS_2_ use *in vivo* and allows for the controlled release of ATE. To our knowledge, this is the first demonstration of a functional MoS_2_-based TDDS. The promising results showed herein warrant further studies to evaluate the *in vivo* application of JR400-MoS_2_ NPs based TDDSs.
